# Trade Commission Acts on Salvarsan Patent

**Published:** 1917-12

**Authors:** 


### TRADE COMMISSION ACTS ON SALVARSAN PATENT

The Federal Trade Commission today entered orders for licenses to three firms to manufacture and sell the products heretofore known under the trade names of "Salvarsan," "606," "Arsenobenzol," "Arsaminol," patent rights which have been held by German subjects. The orders for licenses are to acceptance and agreement by the licensees to the stipulations made by the Commission. Upon such acceptance and agreement , licenses Nos. 1, 2, and 3, will be formally granted by Secretary L. L. Brancken, acting for the Commission.

Hereafter, this important drug will be manufactured and sold under the name of "ARSPHENAMINE."

The Trade Commission's action was taken under Section 10 of the Trading With the Enemy Act under direction of Commissioner Fort, upon recommendation of C. H. McDonald, Edward S. Rogers, and Francis Phelps, in charge of granting such licenses. The Public Health Service has prepared rules and standards for the manufacture and testing of "Arsphenamine." and will supervise its manufacture, authority having been conferred on the Public Health Service by the Secretary of the Treasury, and the observance of the rules and standards become a condition of the license.

The three firms which will be hereby permitted to manufacture and sell "Arsphenamine," are Dermatological Reearch Laboratories, Inc., of New York, and Farbwerke Hoechst Company (Herman A. Metz Laboratory), of New York. The original patent for manufacture of what has heretofore been known as "Salvarsan," etc., was issued to Paul Ehrlich and Alfred Berheim, German subjects and assigned to Farbwerke Vormals Meister, Lucius and Druning of Hoechst of the Main, Germany.

The supply of the drug now licensed to be made in America, up to 1915, was almost exclusively obtained by importation from Germany. It is at present the only known specific for virulent blood poison. From the outbreak of the war importation became more difficult.

Before the war began, the patent drug was sold at $4.00 per dose, which is approximately 3,500 per pound, and speculatively it has brought as high as $35.00 per dose. While the price of the product is not fixed at this time by the Commission, the right to fix prices is retained, and a price of $1.00 per dose to the Army and Navy, $1.25 per dose for hospitals, and $1.50 per dose for physicians, are the prices at which some, at least, of the licensees have stated that they intend to offer the licensed drug.

The enormous shortage of supply on this important product will immediately be relieved, and the article placed in the hands of the Government, the hospitals and the medical profession at a price lower than ever before.

